# Asian Elephant (*Elephas maximus*), Pig-Tailed Macaque (*Macaca nemestrina*) and Tiger (*Panthera tigris*) Populations at Tourism Venues in Thailand and Aspects of Their Welfare

**DOI:** 10.1371/journal.pone.0139092

**Published:** 2015-09-25

**Authors:** Jan Schmidt-Burbach, Delphine Ronfot, Rossukon Srisangiam

**Affiliations:** 1 World Animal Protection, Thailand office, Bangkok, Thailand; 2 University of Exeter, Exeter, Devon, United Kingdom; Tulane University, UNITED STATES

## Abstract

This study focused on determining the size and welfare aspects of Asian elephant, pig-tailed macaque and tiger populations at facilities open to tourists in Thailand. Data were gathered from 118 venues through direct observations and interviews with staff. A score sheet-based welfare assessment was used to calculate scores between 1 and 10, indicating each venue’s welfare situation. Factors such as freedom of movement for the animals, access to veterinary care, environmental noise quality, hygiene standards and work intensity were included in the score sheet. 1688 elephants, 371 macaques and 621 tigers were found at the venues. 89 venues exclusively kept elephants, 9 designated ‘Monkey schools’ offered macaque shows, 4 venues kept primarily tigers, mostly for petting and photo opportunities, and the remaining venues kept a mix of these animals. A strong imbalance in female to male gender ratios was recorded with about 4:1 for adult elephants and 1:4 for adult macaques. Severely inadequate welfare conditions were common, with 75% of macaques and 99% of tigers being kept at venues with scores less than 5. 86% of elephants were kept in inadequate conditions at venues with scores between 3 and 5, but a significant number of venues with scores above 5 were found. 4.6% of elephants were provided commendable conditions, reaching assessment scores of 8 and above. 71% of venues did not offer any sort of education about animals to visitors. This study is the first to assess welfare aspects of captive wild animals at tourism venues across Thailand. It concludes that significant concerns exist about the welfare of wild animals in the tourism sector of Thailand. Urgent attention needs to be given to address these concerns and prevent further suffering. But also to ensure the demand for wild animals doesn’t have a negative impact on wild populations.

## Introduction

Thailand is home to (or borders with) a large area of natural habitat for many wild species which are partially managed in protected areas, including species which are listed as endangered by the International Union for Conservation of Nature (IUCN) [1]. International concerns have been raised regarding various conservation issues with Thailand being identified as a source and transit country in the international wildlife trade[2–4]. Thailand, as a prime tourist destination in Asia, attracts well over 15 million visitors annually [5]. Over the years Thailand’s tourism industry has continued to maintain and promote attractions using wild-caught or captive-bred wild animals with little regulation or monitoring [6–8]. While in the past this wildlife entertainment industry was of smaller scale, potentially high profit margins for some venues have led to an increasing development of this sector. No database exists accounting for the numbers of animals at such venues or for the welfare conditions they are kept in. Due to missing accountability and limited regulation, valid concerns exist that the demand for wild animals through tourism is having a negative impact on the conservation of wild species and that the existing captive wild animals are suffering through inadequate husbandry conditions [9].

Three species are most commonly used as attractions for tourists in Thailand: Asian elephants (*Elephas maximus*), macaques (mostly *Macaca nemestrina*) and tigers (*Panthera tigris*). The use of elephants and macaques stems from the traditional role these animals played in South-East Asian rural communities. Macaques are used for coconut harvesting, especially in the southern provinces [10]. Elephants have been used for more than 4,000 years for transport of goods, logging, war or religious ceremonies[11]. Apart from a small amount of captive bred animals, macaques as well as elephants have mainly been sourced directly from the wild and thus cannot be considered domesticated animals [12,13]. In the case of elephants, discussions are ongoing about what the appropriate term for elephants kept by humans should be[12–15]. In light of the intrinsic cultural tradition of keeping elephants, the complex relationship between elephant keepers and elephants and the varying definitions of domestication it is suggested to recognize the continuous nature of the transitional process from wild to domesticated, rather than limiting terminology to these two extreme outliers [13,16]. However, distinguishing between taming and domesticating is essential for the role of elephants and macaques in the entertainment industry. According to Russell [15], taming is a relationship between a particular person and a particular animal without long-term effects beyond the lifetime of that animal; while domestication is a relationship with a population of animals that often leads to morphological and behavioural changes in that population. It is beyond the scope of this paper to discuss these definitions in depth. We will use the term ‘captive elephants’ as in the authors’ opinions this reflects most closely the situation of the animals, given their lack of domestication and adaptation to life in human communities.

Captive elephants in Thailand are required to be registered with the Ministry of Interior within 8 years of age; in 2012 approximately 2,700 elephants were listed as registered [17]. Exploitation of this registration scheme occurs and frequent incidences of wild elephants being poached and illegally traded across the Burmese-Thailand border to be laundered into the elephant tourism industry have been reported recently, sparking serious conservational concerns [18].

Tigers have been bred in captivity in Thailand for at least 20 years, with one of the largest venues heavily promoting their successful breeding program at the site. However, due to poor or absent scientific management of breeding efforts the tigers are of no significant genetic value for species conservation purposes [3]. Whether farming of tigers can actually be a tool for conservation is heavily disputed, with most authors highlighting the risk of sustaining a demand for cheaper wild animal-derived products [19] or the opportunity for laundering illegal wild products [20]. Conversely, other authors suggest that farming of tigers should be considered in certain circumstances, e.g. in case of inadequate protection of wild tigers in tiger habitat countries a farming option may decrease pressure on the wild population [21].

With few exceptions from an ethical and welfare based point of view many believe that the ideal environment for a wild species is its natural habitat. During hundreds of generations, wild animals have adapted to their environment and to its specific challenges. It is commonly acknowledged that in captivity, the welfare of a wild animal may primarily be dictated by how well the husbandry conditions resemble its natural environment, thus catering fully to its physiological and psychological species-specific needs[22]. Addressing all of the animals’ needs should be the goal of any institution keeping wild animals, be it rescue centre, public zoo or private venue. Meeting all of those needs is next to impossible in a captive environment, not only because it is difficult or too expensive to replicate the wild environment but also because we only have a rudimentary idea of the actual needs of most species. Inadequate conditions for wild animals, such as limited space, artificial substrates, reduced social interaction or missing stimulation of species- specific behaviour will lead to physical or psychological damage, manifesting itself in injuries or behavioural problems such as stereotypies, self-mutilation or aggression [23]. Many progressive zoos and sanctuaries nowadays try to address these issues by designing large enclosures which closely resemble natural habitat and by introducing extensive behaviour enrichment programs to keep the animals stimulated [24]. Although these are important improvements for the captive animal, they cannot fully replace a wild environment. The conditions for wild animals at private tourism venues in Thailand have only been sporadically evaluated in the past. The lack of husbandry guidelines and currently limited animal welfare legislation for captive wild animals in Thailand indicates a pressing need to assess the situation in more detail to gain a better understanding of the scope and severity of animal welfare problems in these venues.

This study aims to create a baseline estimate of the numbers of animals kept at entertainment venues in Thailand, excluding zoos. Furthermore, an attempt was made to assess the welfare conditions these animals face through a simple yet practical scoring method. It is hoped the results of this study will influence future decision-making by tourism and elephant industry stakeholders and the Thai government in order to reduce animal suffering. The results may also contribute to discussion of the impact a demand for wild animals has on the *in situ* conservation of these species.

## Materials and Methods

During the survey, 118 wildlife entertainment venues throughout Thailand were identified, located and visited between May and December 2010. As no database exists listing these enterprises, venues were located partly by researching tourist reports on the internet as well as by directly tracking them locally by driving along major roads and investigating offers by local tour agents in day trip distance of all major tourist destinations. These tourist destinations included the greater Phuket area, Phang Nga, Krabi, Khao Lak, Ko Lanta, Ko Samui, Ko Phangan, Suratthani, Hua Hin, greater Bangkok area, Chonburi, greater Pattaya area, Ko Chang, Surin, Kanchanaburi, Ayutthaya, Sukothai, greater Chiang Mai area, Mae Rim, Pai, Chiang Rai and Mae Hong Son. Each venue’s exact location was stored using GPS units Garmin GPSMap 60 CSx or as backup a Nokia C5 with GPS.

Venues which were open to walk-in visitors were visited during regular opening times without advance announcement. At venues where this was not possible the visit was announced in advance to the management. It is the authors’ opinion that advance announcement of the visits in these cases will unlikely have had any significant impact on the scoring, as the husbandry situations could not have been altered on such short notice. During each visit, venue staff was aware of the presence of the researchers, and full anonymity of the collected data was assured to address some venues’ concerns about public resentment of inadequate conditions. Thus, names or coordinates of venues will not be shared in this publication. Data on animal numbers and animal management procedures were collected through unstructured interviews with staff at the venue, supported by verification through direct observation. No information was collected where explicitly prohibited or considered illegal. Private land of visited venues was only entered after receiving permission from staff or ticket offices and no confidential data or animal samples were collected. Rapid assessment of body condition, gender (of elephants and macaques; not tigers due to the large numbers in some of the venues and inaccuracy of defining gender from a distance) and visible health issues was carried out through direct observation on a best-effort basis since close access to all animals was not always possible. Elephants below an estimated age of six years were recorded as juvenile. Age estimation was done according to body size and maturity signs, as well as information from the venue staff.

Based on the observations at each venue, scores were attributed to several husbandry key factors influencing the welfare of each species. These key factors included mobility, hygiene and shelter, environmental noise quality, naturalness of environment, social interaction, diet, entertainment intensity and animal management. For elephants, hygiene and shelter were assessed individually from each other as animals daytime routine would often lead to the animals spending time in non-shelter areas where hygiene may still play an important role. The selection of these criteria is based on the established concept of the Five Freedoms for farm animal welfare, stating that the freedom from hunger and thirst, discomfort, pain/injury/disease, fear and distress, and the freedom to express natural behaviour should guide considerations to promote good welfare [25]. Scores for each husbandry factor were allocated along a 5-point scale, where severely inadequate conditions scored lowest, increasing incrementally with quality of the factor. Efforts were put into closely approximating an interval-level measurement of husbandry conditions by creating equivalent distances between scale scores for each key factor (S1 Table). Eventually, for each venue the scores of all welfare-impacting factors along the scale were added and converted into a final rating score between 1 and 10 by FS = (x/xmax)*9+1, where FS = final rating score, x = husbandry score, xmax = maximum achievable husbandry score. This rating score can only reflect the daytime husbandry conditions as data was not collected for night time conditions, which in some cases may differ from the day time conditions.

Additional to the score sheet assessment, data of specific husbandry characteristics was collected for 27 categories (S2 Table). Value definitions for each category were used to ensure uniform assessment quality (S3 Table). This also included basic behavioural observations, carried out over a 5 minute period of each group of animals and recording the percentage of animals showing stereotypic behaviour for each assessed venue. The observer’s location was chosen so that the animals’ behaviour would not be significantly impacted by the presence of the observer. All observations were recorded photographically and the collected data stored for subsequent evaluation.

For a few analyses, the elephant venues were grouped into three regional groups; ‘North’ being every venue located north of Phitsanulok, ‘Central’ between Phitsanulok and Hua Hin and ‘South’ every venue south of Hua Hin. The three regions have significant economic and cultural differences as well as distinctively different attraction for tourists, possibly impacting the results of the survey.

Correlation analyses were made using Spearman’s rank correlation coefficient method. For this purpose, approximate interval-level measurement qualities of the designed scale was assumed. Values with p<0.01 were considered significant.

Educational material was assessed according to availability and defined as ‘Basic’ if posters or boards with information about any animal related issue were displayed, if videos were shown on a TV or audio recordings played. ‘Comprehensive’ was true if dedicated staff members were observed addressing visitor groups or the groups were being directly asked to pay attention to specific educative media, such as screened films, audio recordings, brochures, posters, etc.

## Results

In total, 1688 Elephants, 371 Macaques and 621 Tigers were found at the venues visited. By far the largest proportion was elephant venues: 106 of the 118 venues kept elephants, 89 exclusively. At one elephant venue a welfare assessment could not be carried out due to limited access. Thus 44 elephants and one venue are not included in the welfare analysis, reducing the total of elephants in the assessment to 1644. Macaques were kept at 21 venues, 9 of which were designated ‘Monkey schools’, focusing mainly on macaque shows and training. Without exception *Macaca nemestrina* species were used by these ‘Monkey schools’. The remaining 12 venues kept macaques in addition to other wild animals, using also species of Macaca fascicularis (48 animals) and Macaca arctoides (14 animals). At 10 venues tigers were kept, with 5 of these venues focussing primarily on this species, keeping more than 30 tigers. A few venues were found to have started operating at several locations, but most venues were enterprises at only one location.

**Fig 1:**
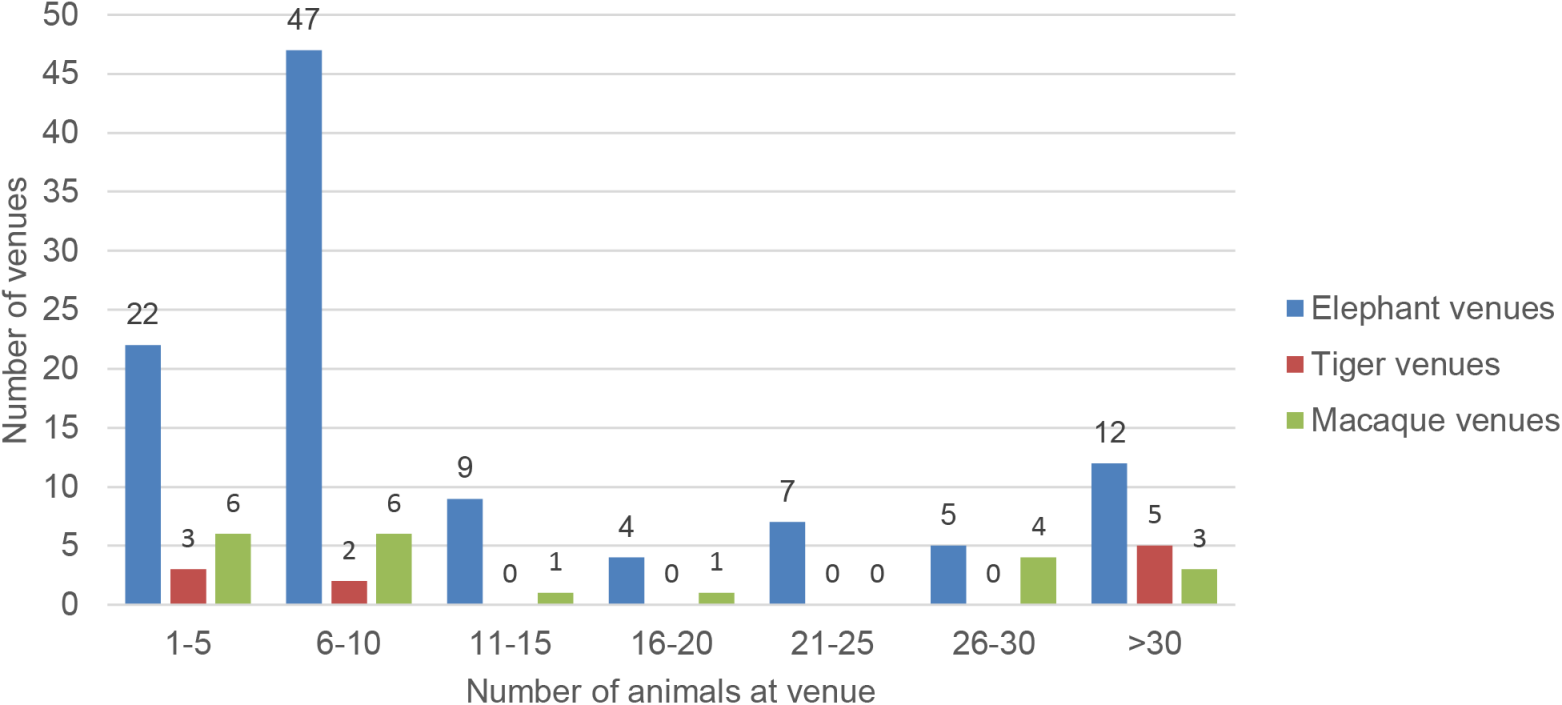
Sizes of venues based on animal numbers. Grouping venues based on the numbers of elephants kept on site at the time of the assessment, shows more than half of the elephant venues being of smaller scale. Most venues were keeping between 6–10 elephants, followed by small scale venues with 1–5 elephants. At the other range of the spectrum 12 venues housed more than 30 animals at their premises. Tiger and macaque venues showed concentrations of venues at either end of the scale but with very few medium sized venues.

Of the 106 venues that housed elephants, the majority were small to medium sized enterprises: 65% of venues had between one and ten elephants, accounting for 26% of all captive elephants. 36% of elephants were housed at the eight largest venues, with 200 elephants being housed at one single government-run centre. These few large venues had a significant impact on mean herd size of venues (Mean = 15.92 Standard deviation = 23.41 and Median = 9, Fig 1).

Of all assessed animals, 70% of all adult elephants in the venues were female and only 18% were male, the remainder being juveniles for which gender was not recorded. This is approaching a ratio of 4:1 for adult females, more imbalanced than in western zoos (3:1)[26]. In macaques, the opposite situation was observed: 67% of the animals were adult males and only 17% adult females with the remainder being juveniles. In some monkey venues, captive breeding was carried out but it was unclear what happened to female offspring, as even in those venues the majority of adult animals were males. It was not feasible to collect accurate gender specific data for tigers in this study.

### Welfare situation

Of the 117 assessed venues, only 12 (10.2%) reported direct access to veterinary care, either through a full-time vet employed by the facility or through a contracted veterinarian. Most venues would thus rely on medical care provided by their staff or on long transport routes of at times several hundred kilometres to appropriate veterinary facilities in case of emergencies.

**Fig 2:**
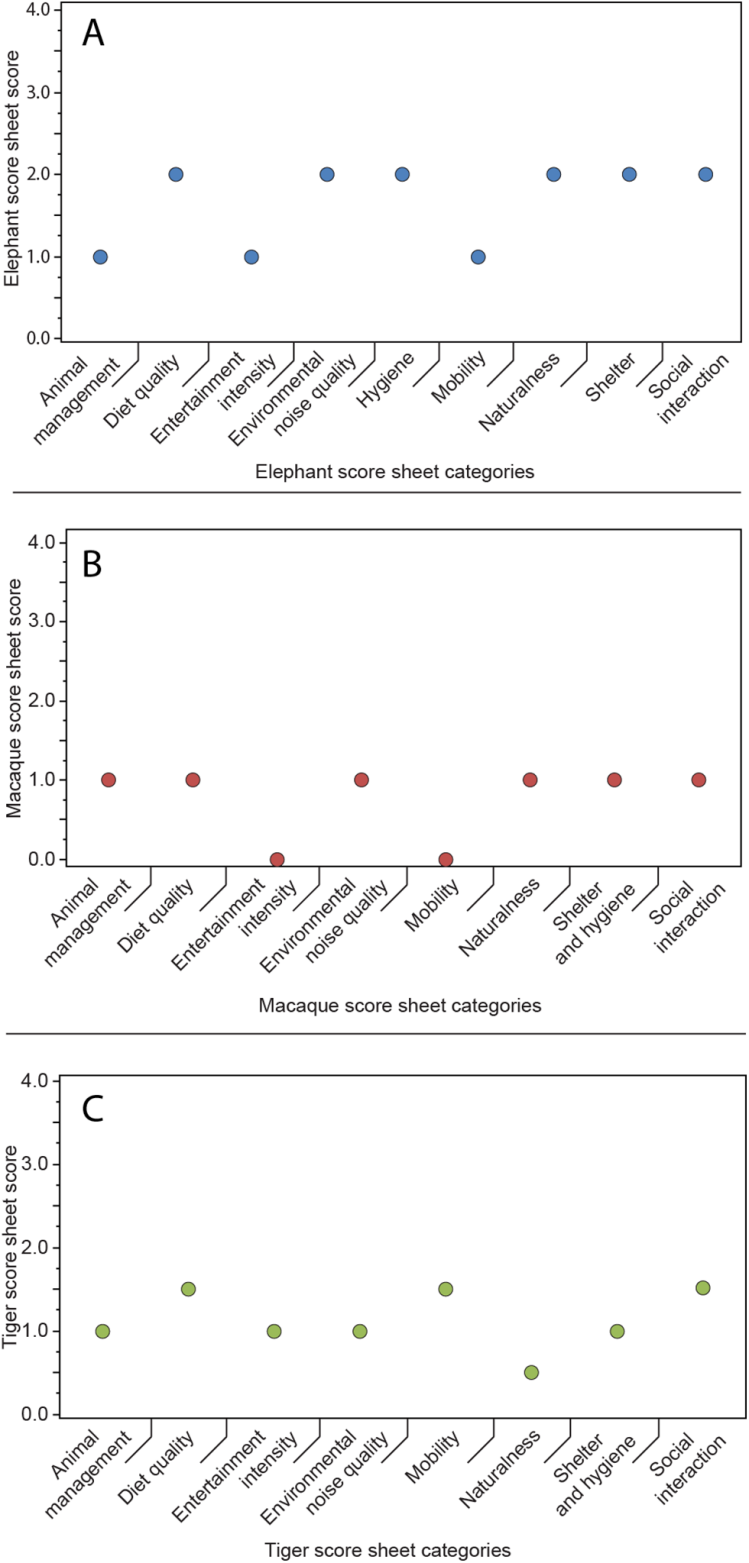
Medians for each score sheet category and species kept at the assessed venues. The figure shows lowest score medians for ‘Mobility’ and ‘Entertainment intensity’ for macaques and elephants, and for ‘Naturalness’ for tigers. While all medians range low on the scale, elephant scores are slightly better than tiger or macaque scores due to a few venues implementing better husbandry standards.

In total, 1422 elephants (86% of all assessed elephants, n = 1644) at 88 elephant venues were kept on short chains during the day except when used in tourism activities, 421 elephants (25.6%) were kept on concrete ground, and 563 elephants (34.2%) at 40 venues were kept separated from each other, not allowing direct social contact. At night most venues claimed to move their elephants into fields or forest where they would be chained on longer chains in a more natural environment but still kept isolated from each other. 95 venues (90.4%, n = 105) offered elephant trekking, 30 venues (28.5%) included elephant shows and 7 venues (6.6%) offered one-day elephant keeper training, usually called ‘Be-A-Mahout’ or similar. 5 venues (3.3%) did not use their elephants for any entertainment activities. Diet composition at the venues most commonly included pineapple leaves (74.2% of venues), followed by grass or hay (63.8%), banana stems/leaves (44.8%) and sugar cane (23.8%). Pineapple leaves were especially popular in venues in southern Thailand, likely due to the more common pineapple agriculture and cheap availability of these thick leaves. 20% of venues reportedly only provided one type of food to the animals. Across all elephant venues the score-sheet categories ‘Mobility’, ‘Entertainment intensity’ and ‘Animal management’ were among the lowest ranking of all assessed categories (Fig 2A and S4 Table).

**Fig 3:**
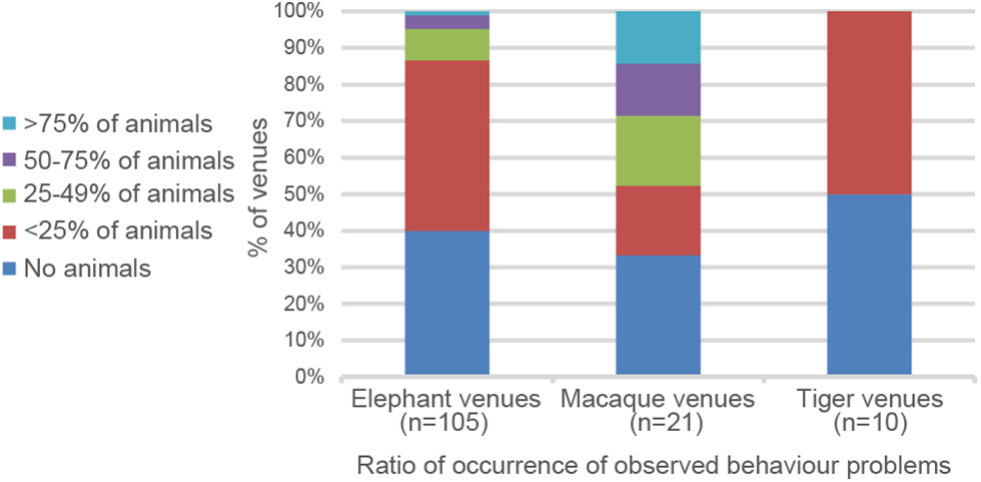
Occurrence of behaviour problems at each assessed venues. The percentage of animals with observed behavioural problems at each venue and the number of these venues give a rough estimate of the frequency of behavioural problems, such as stereotypic behaviour. Care must be taken in interpreting these findings as observations were only possible during the relatively short time frame of the visits to each venue.

The survey design did not permit detailed assessment or evaluation of stereotypic behaviour, but such behaviour was recorded if it was observed at the time of the visit. Thus these data can only serve as a rough estimate of the situation. Behavioural problems, mainly weaving and pacing, were observed in animals at 63 venues (60%). Incidences ranged from a few affected animals at each venue in the majority of cases, to five venues with more than 50% of animals showing stereotypic behaviour. At 42 venues (40%) no stereotypical behaviour was observed during the visit (Fig 3, Elephant venues).

At macaque venues, movement of animals was severely restricted with all but one venue keeping animals in small cages or on short leashes. More specifically, 16 venues (n = 21) provided cage space of between 1–10 sqm per animal. Frequently macaques were kept on leashes within cages, this allegedly being a means of training the animals not to get entangled in the leash when harvesting coconuts. 15 venues kept most of their animals isolated from each other, not allowing free direct social contact. Visual contact was possible in most cases. In contrast to the management of elephants, this caged or leashed condition was maintained for 24 hours a day, except when the animals were used for tourism activities. Shows were offered at 18 venues, with 10 repeating shows more than 3 times daily. Across all macaque venues the score-sheet categories ‘Mobility’ and ‘Entertainment intensity’ ranked lowest of all assessed categories, similar to the situation in elephants (Fig 2B and S4 Table). Behavioural problems were observed in 14 venues (67%) with 6 venues having more than 50% of their animals affected (Fig 3, Macaque venues). The most common behavioural problem observed was pacing (at 9 venues), followed by apathy (6), weaving (5) and self-mutilation (4).

Tigers were usually kept in caged enclosures, with 4 venues (n = 10) chaining a few tigers on platforms for visitor interaction. 50% of venues provided barren, non-enriched cages of 10–50sqm, shared on average by two tigers. A few venues provided concrete enclosures for groups of tigers with between 2 and 12 individuals. Access to fresh water was not available in 3 venues and only for few animals in 4 (n = 8). For two venues data on fresh water availability was inconclusive and was discarded. 8 tiger venues provided photographic posing activities for tourists and 5 offered tiger cub feeding using cubs removed from their mother–in most cases apparently permanently. Behavioural problems were less common than in other species surveyed with no observed problems at 50% of the venues and fewer than every fourth animal performing stereotypic behaviours in the remaining 50%.

**Fig 4:**
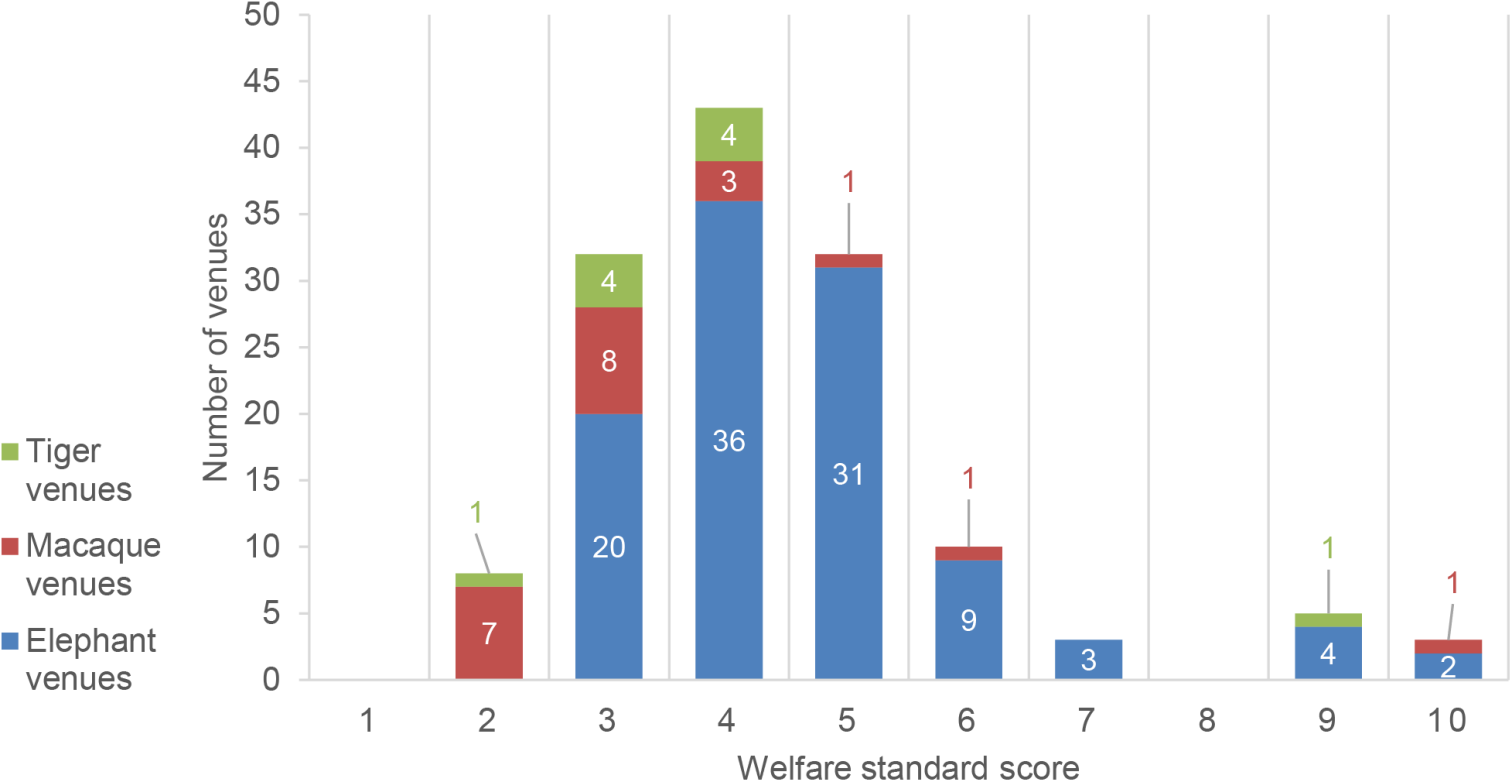
Distribution of venues according to their daytime welfare score (higher score = better welfare). Elephant venues peak around the score of 4, representing inadequate conditions, with a gradual decrease to score 7 and a few venues showing better welfare with highest scores. Macaque venues rated worse with a peak around the score of 3 and a large number of venues even only achieving a score of 2. The few tiger venues fell between scores 3 and 4 with one good exception for a single tiger. The numbers of venues keeping tigers, elephants or macaques were plotted according to the animal welfare score calculated by this study for each venue.

**Fig 5:**
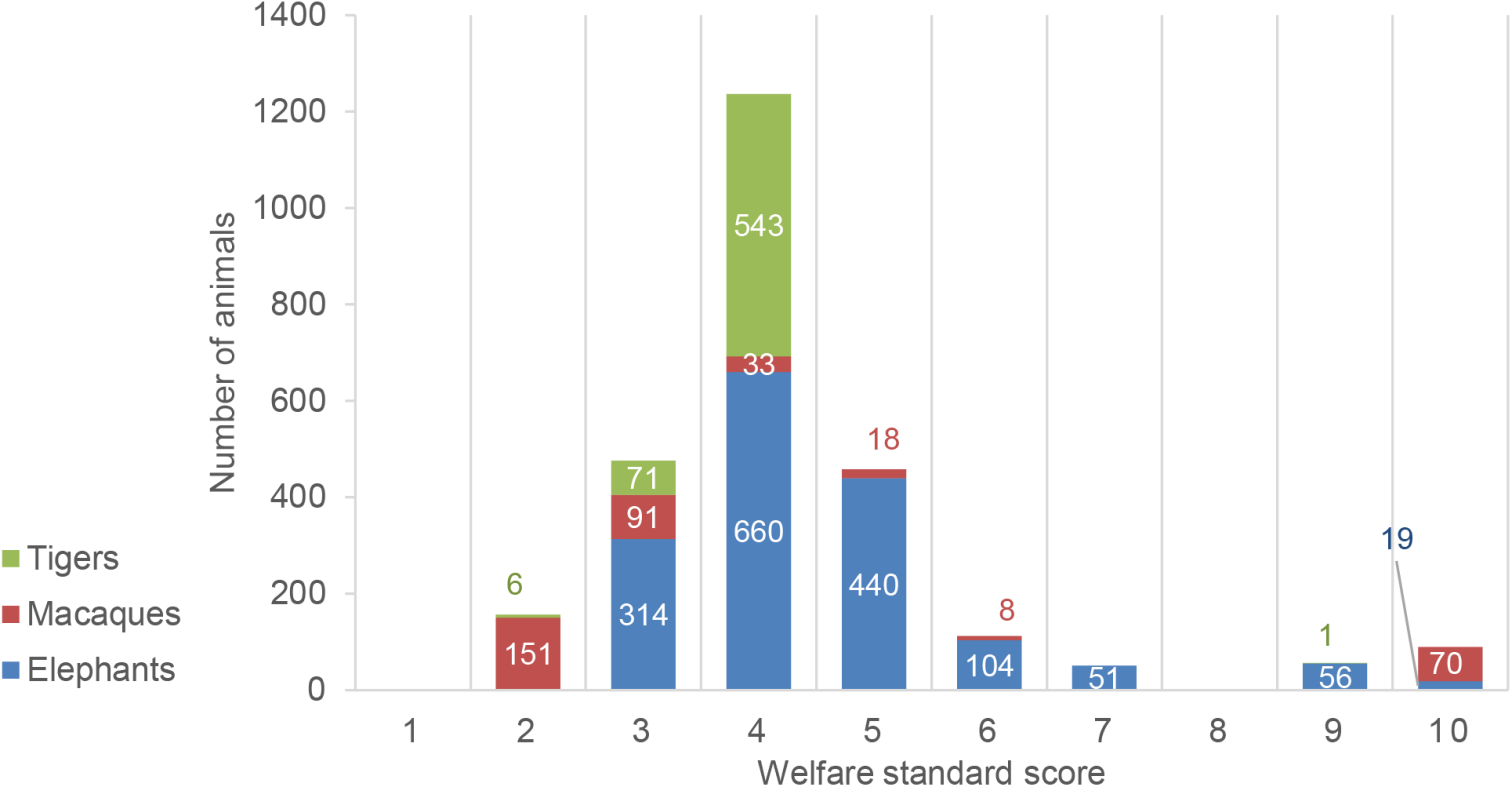
Distribution of animals according to their daytime welfare score (higher score = better welfare). The numbers of tigers, elephants and macaques were plotted according to the animal welfare score calculated by this study for the venues the animals were kept at. The figures largely correspond to the distribution of venues according to their welfare score. Most elephants and tigers are kept in conditions reflecting a score of 4, while the majority of macaques are kept at even lower quality venues with a score of 2.

Approximately 90% of tiger and 85.7% of macaque venues received welfare assessment scores of 4 or less, suggesting severely inadequate conditions are common for these species. These scores reflect the observations made during this study including the use of concrete pits or cages for tigers, cages or short leashes for macaques, impossible or extremely limited social contact between conspecifics for macaques, absence of environmental enrichment, fundamental lack of hygiene, mistreatment by handlers and severe stress or discomfort as a result of show activities or intense interaction with visitors. The single tiger venue with a welfare assessment score of 9 housed only one tiger, therefore 99% of tigers were kept in severely inadequate conditions. A similar situation applied to macaques: the single venue with a score of 10 was not a monkey school but a wildlife rescue facility open to visitors. 70 macaques experienced the best possible captive conditions there. However, 275 animals were housed at venues with scores between 1 and 4, representing mostly severely inadequate conditions (Fig 4, Fig 5).

The situation regarding elephants was slightly better. While the majority of elephants (1414 animals, 86%) were kept in inadequate conditions at venues with scores between 3 and 5, there were a significant number of venues with scores above 5 and 4.6% of venues provided commendable conditions for 75 elephants, reaching assessment scores of 8 and above.

A very clear geographical gradient of animal welfare standards at elephant venues was found when comparing regions. From north to south the proportion of low scoring venues increased and the proportion of higher scoring venues decreased. In south and central regions 92% and 93% of elephants respectively were kept in severely inadequate conditions. In the north only 54% of the elephants faced those conditions.

**Fig 6:**
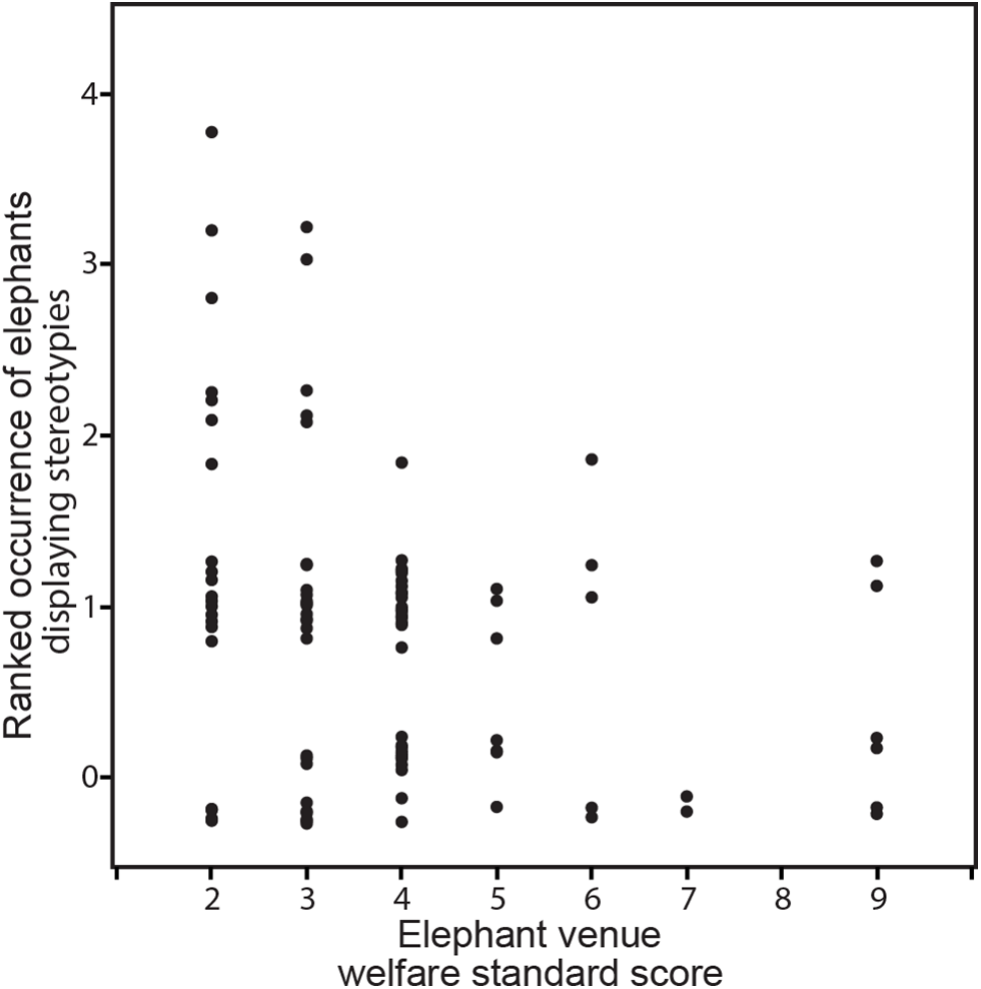
Scatterplot of ranked occurrences of elephants displaying stereotypies at assessed venues against welfare standard scores of these venues. The ranking scale steps are 0 = No animals stereotyping, 1 = <25% of animals stereotyping, 2 = 25–49% of animals stereotyping, 3 = 50–75% of animals stereotyping and 4 = >75% of animals stereotyping. Venues with lower welfare standard scores show a higher occurrence of stereotypies of elephants than venues with higher welfare standard scores.

A Spearman’s Rank correlation analysis was carried out on the percentage of elephants recorded that displayed stereotypic behaviour at individual venues and their final assessment score. There was a significant negative correlation between percentage of elephants performing stereotypies and assessment score (corr(assessment score, stereotypy prop) = -0,34, p<0,001): The higher a venue ranked in this study, the smaller the percentage of observed stereotyping elephants (Table 1 and Fig 6). While this may not be surprising, it could support the suggestion that higher welfare standards may be associated with reduced development of behavioural problems. To validate this observation more research on elephant stereotypic behaviour, other stress indicators and causative factors is required, as the data presented on stereotypies here can only provide an overview. Correlation analyses on behavioural abnormalities for macaques and tigers did not provide sufficient significance of results.

**Table 1:** Spearman’s Correlation Coefficient and P-value for correlations between ranked occurrence of animals displaying stereotypies and the venues’ welfare standard score. For elephant venues a significant negative correlation can be seen, while for macaque and tiger venues the data did not produce significant enough results.

Variable	by Variable	Spearman p	*p*
Ranked occurrence of elephants displaying stereotypies	Elephant venue welfare standard score	-0.3429	<0.001
Ranked occurrence of macaques displaying behavioural abnormalities	Macaque venue welfare standard score	-0.1105	>0.1
Ranked occurrence of tigers displaying behavioural abnormalities	Tiger venue welfare standard score	-0.3343	>0.1

### Education of visitors

71% of venues did not offer any kind of education and only 6% offered comprehensive education. Almost no visitors were observed to use the education facilities at venues that offered ‘basic education’. Some of the larger venues provided discounted access to school groups and could potentially provide education about wild animals. Unfortunately, this was not found to be the case. Some of the venues were attended by hundreds of young school children as part of educational school trips. Usually these groups would observe offered wild animal shows including elephants balancing on tight ropes, acting as goal-keeper or shooting darts at balloons, raising questions about the educational impact on the children Noise volume was typically extremely loud through amplified music and narration of the show. These shows of wild animals for young children and adults alike contained no educative value on the natural origin, behaviour and critical status of these animals. Impact of such experiences on the attitudes and behaviour of child audiences in relation to wildlife conservation and animal welfare are of concern and would benefit from further research.

## Discussion

Some peculiarities were observed in the gender ratio of elephants and macaques. However, the 4:1 female-to-male elephant ratio found at tourism venues may differ for the whole population of captive elephants in Thailand, depending on the number of male elephants outside of these venues. A previous assessment at elephant camps in Northern Thailand measured a female-male ratio of 2:1 with an increase of this ratio with age of the animals [27]. Male elephants are less predictable and harder to control than females due to their greater size and their regular musth intervals–periods where males undergo hormonal changes often linked with increased aggression [28–30]. Thus venue managers may prefer to keep female elephants to reduce risk of injury to visitors or property. The ongoing efforts to encourage captive breeding of elephants in Thailand may result in a shift towards a more balanced gender ratio in the future. This will pose challenges for elephant management at venues, since management of elephant bulls is significantly more difficult. This challenge may result in an increasing number of bulls being under extreme restraint or being used for logging activities, raising concerns about their welfare and care through overworking and limited access to veterinary care in border regions. Greater demand from tourism venues for females and especially infants may also influence illegal trade. As a result of recently highlighted on-going illegal elephant trade across borders into Thailand, often involving juvenile female elephants, monitoring and strict prevention of these activities are critical for wild elephant survival [9,31,32]. With populations of wild elephants in Thailand estimated to be around 1,000 and in Myanmar round 1,600 animals any demand for live traded elephants may have detrimental impact on these wild populations [33].

The recorded higher ratio of male macaques may stem from owners’ preference for males as in personal communication with macaque owners they were perceived to be stronger and having greater endurance when used for coconut harvesting. Most macaques are reported by owners as captured directly from the wild and the observed unbalanced gender ratio may support these reports. Alternatively, unwanted captive bred females may be traded for other purposes. The magnitude of the trade in macaques for tourism purposes seem to be of little conservation impact due to the low numbers of animals kept for this purpose. The potential coexistence of this trade with other trade routes however may need further investigation, since other macaque subspecies populations, especially *Macaca fascicularis*, have been reported to have rapidly declined over the past ten years due to increased hunting for export to be used as lab-animals [34].

Gender data was not collected for tigers during this assessment. The frequent practice of captive breeding of tigers observed in this study and the reportedly stable populations at some of the visited venues gives rise to the question of the final destination of the animals bred. A previous investigation alleged involvement in illegal tiger trade at one of the tiger venues in Thailand [6]. In 2012 a driver was arrested near the Lao border, illegally transporting 16 tiger cubs from an unknown origin in Thailand [35]. Also a recent investigation into tiger farming in Thailand and Laos suggested links between tiger tourism venues in Thailand and a Lao tiger farm. Two of the investigated facilities in Thailand were breeding together about 100 tigers a year, strongly underlining the need for better transparency regarding purpose of these breeding activities and destination of the tigers [36].

Rees’ (2008) study on elephant group sizes in zoos revealed a global mean group size of around 4 elephants (Mean = 4.34, Standard deviation = 6.83) [26]. The present study reveals larger mean group sizes at elephant venues, coupled however with very large variation as a result of there being a few venues with very high numbers of elephants (Mean = 15.9, Standard deviation = 23.4). It is important to understand that in most venues the elephants are not kept as a group but as individuals, denying access to the positive effects of group interaction for these social animals. Thus in terms of impact of herd size on the welfare situation it is difficult to compare this study with Rees (2008). The vast majority of elephants were kept chained for most of the day and tied to ropes or long chains during the night. This restraint is reportedly used to help ensure safety of handlers and visitors and to limit damage to property or crops by free ranging, unsupervised elephants. It is suggested that keeping elephants unchained under more and better supervision by elephant handlers may allow for more freedom and social interaction, increasing elephants’ welfare without incurring much more expense by the venue. As there may only be limited scope for improving the welfare situation in many venues, these results underline the importance of debating whether elephants can be kept adequately in captivity at entertainment venues.

Welfare condition scores of 4 or lower represent extremely inadequate conditions for macaques, tigers and elephants. Depending on species, between 50–90% of all venues received such low scores in this study, suggesting that the situation needs to be addressed urgently. The existence of several elephant venues with assessment scores above 4 suggests some awareness about, and availability of, better husbandry methods. The few venues with scores above 8 stand out well above the rest, however, their standards may not be easily expanded to include all elephants, due to the comparatively high costs of establishing and maintaining such facilities. However, an increase of tourist demand for high welfare facilities may drive improvement of other venues to follow these examples, while initiating a phase out of the worse ones.

The welfare assessment methodology introduced in this study was designed to be used during relatively short visits to venues, housing a variety of species. It aimed to determine environmental and husbandry conditions and, based on these conditions, provide a strong indicator for the welfare situation of individual animals. A compromise had to be made between level of detail of the assessment, interference with venue management and time investment by researchers. Subjective variations in scoring were minimised by giving specific descriptions for most scores in each criteria. However, at times the assessors would face conditions that would meet parts of the description of one score and other parts of an adjacent score in the same criteria. In these few cases it was left to the assessor’s best judgement to select the most applicable score, primarily based on which factor affected more animals. Thus this rapid assessment methodology has its limitations, but also enables researchers to gain welfare insight in situations that otherwise could not be assessed. The assessment methodology has not been validated and as such it must be pointed out that the results should only serve as indicators for the welfare situation at venues in Thailand. However, the selected key criteria, such as Hygiene, Noise, Diet, Shelter, Mobility, etc., are well recognized to be directly impacting animal welfare, as they are directly linked with the recognized principle of the Five Freedoms [25]. Scorecard based assessments are common in the field of animal welfare and have been applied from assessing individual animal’s body condition to drawing conclusions on the overall welfare situation for animals in complex farming situations [37,38]. It is important to note though that the assessment methodology applied in this study focusses primarily on provisional factors impacting welfare, such as husbandry and animal management, while lacking assessment of welfare outcomes, such as individual animal’s fitness. Assessing both provisional factors and welfare outcomes would be ideal [39], however, was not feasible due to the limited assessment time at each venue and restricted access to observe individual animals.

Thus this assessment methodology differs from that of an earlier study on welfare and health determinants in Asian elephants which put a stronger focus on individual elephant health and owner knowledge and extrapolated from this to the larger population [40]. This earlier study concluded that normal appearance of elephants may not necessarily guarantee good welfare. In the case of elephants, the frequent observations of poor animal welfare in the present study represents daytime husbandry standards at the venues, as conditions during the night were not evaluated in detail. It is believed, however, that the main welfare concerns relating to elephants in this study, limited free social interaction between elephants and restricted movement, are also relevant to night conditions. This study did not directly measure stress in the animals; validation of the assessment methods used by measuring chronic stress levels or more thorough behavioural observations would be useful future research, provided reliable stress measurement tools and methods are available in the species concerned.

The regional variation in animal welfare conditions provided at elephant venues may be explained by a combination of observations. Firstly, some of the regional ethnic groups in the northern and central-eastern areas are renowned for their long elephant capture and management tradition. It is likely that this knowledge manifests itself in generally better husbandry conditions. Also, venues in the north are, to a greater degree, permanent camps while in the south many camps seem to open and close depending on the season. A permanent camp might present benefits for elephant management, as facilities are more likely to be of better quality [40]. Last but not least, visitors travelling to the northern regions might have different expectations of an experience with elephants than visitors in the south. While the southern regions are mainly famous for their beach and ocean environment, the north is popular for cultural diversity and natural forest. Visitors to the north are likely to expect a more natural experience when visiting an elephant camp than those visiting the south. Thus elephant camps may try to cater to this expectation.

## Conclusion

The results of this study give cause for significant concerns about the welfare of elephants, tigers and macaques at tourism venues, as well as concerns regarding the potential negative impact of the industry on the conservation of these species in the wild. The conservational value of captive populations of these wild animals need to be carefully reviewed in light of these venues sustaining a demand for wild-caught individuals and causing suffering through inadequate conditions. The imbalanced gender-ratios of elephants and macaques should be monitored and investigated more thoroughly to evaluate the ratio of animals originating from the wild, as should the final destination of tigers bred at tiger venues, in order to respond to allegations by various authors of involvement in the illegal tiger trade. The welfare situation for the vast majority of animals kept at venues accessible to tourists is deeply worrying and the husbandry conditions must be improved as a matter of urgency. The existence of a few higher welfare quality venues for some of the captive elephants must be positively acknowledged and will hopefully serve as a model for replication to benefit the majority of elephants. Developing appropriate husbandry guidelines for wild animal species coupled with regular monitoring of compliance with these, and efforts to increase animal welfare awareness of tourists, may lead to an increase of the demand for better welfare facilities. However, simultaneously it must be considered to initiate a transition away from conventional wildlife entertainment towards establishing a more sustainable tourism model that protects wild animals in their natural habitat and preserves Thailand’s wild animal heritage. With Thailand’s attraction for international tourists this would be an opportunity for Thailand’s tourism industry to take a lead in demonstrating a successful marriage between wildlife preservation and tourism. Especially for the benefit of captive and wild elephants in Thailand it seems crucial that the government and tourism industry explores and adopts policies that safeguard the welfare of these captive animals and prevents capture of wild elephants. The registration procedure for young elephants must urgently be addressed to close existing loopholes which potentially allow for laundering of wild-caught elephants into the tourism industry. The exploitation of these loopholes was recently documented for 79 to 81 elephants poached in the wild in Burma and illegally traded into Thailand to be laundered into the tourism industry [18]. In the long run the demand for captive elephants must be eliminated to prevent further cruelty and preserve elephants in the wild. Lastly, this research recorded a large number of tigers at tourism venues, partially being used for breeding. Establishing an increased transparency into the purpose and scale of these breeding activities and the eventual destination of the animals will help to ensure compliance with CITES regulations. In general, further research on tourist attitudes regarding animal welfare, as well as welfare conditions and stress experienced by captive wild animals used for tourism, along with identifying economically feasible and humane solutions, is required. This will benefit efforts to decrease suffering of wild animals used for tourism and hopefully lead to wild animals remaining in the wild where they belong instead of being kept in captivity for entertainment.

## Supporting Information

S1 Table Score sheet for elephant (A), macaque (B) and tiger (C) venues for rapid welfare assessment. The description for each score was used solely as guidance by the assessor. In case of situations where a venue showed a combination of different score descriptions, it was the assessor’s task to decide on a representative score based on the intended interval-level quality of husbandry conditions.
(PDF)

S2 Table List of data points and possible answers collected from each venue during the assessment visits. These data points in conjunction with direct observations and photographic records were with the score sheet to estimate a welfare score for each venue.
(PDF)

S3 Table Definitions of terms used in this study. (PDF)

S4 Table Medians of the score sheet scores by husbandry factor and species kept at the venues. The table shows lowest scores for elephants and macaques for ‘Mobility’ and ‘Entertainment intensity’, while for tiger venues lowest scores resulted for ‘Naturalness’ and also ‘Entertainment intensity’. Means are given purely as orientation as the data is ordinal. However, due to the approximate interval-type nature of the used scale, means may give valuable additional insight.
(PDF)
