# Supplementary material for: Asian Elephant (Elephas maximus), Pig-Tailed Macaque (Macaca nemestrina) and Tiger (Panthera tigris) Populations at Tourism Venues in Thailand and Aspects of Their Welfare
Source: PLoS One. 2015 Sep 25;10(9):e0139092. doi: 10.1371/journal.pone.0139092 (PMC4583339; doi:10.1371/journal.pone.0139092)
Supplement: S1 Table — The description for each score was used solely as guidance by the assessor. In case of situations where a venue showed a combination of different score descriptions, it was the assessor’s task to decide on a representative score based on the intended interval-level quality of husbandry conditions. (PDF) [file pone.0139092.s001.pdf]

S1 Table: Score sheet for elephant (A), macaque (B) and tiger (C) venues for rapid welfare assessment. The description for each score was used solely as guidance by the assessor. In case of situations where a venue showed a combination of different score descriptions, it was the assessor's task to decide on a representative score based on the intended interval-level quality of husbandry conditions.

A) Elephant venue score sheet

Category/Score	0	1	2	3	4
**Mobility**	Mostly on short chains + trekking activity	long chains/small pen + trekking	Pen > 100sqm + trekking	Most time of day large enclosure access	Free and unrestricted movement
**Hygiene**	Old faeces + urine present, moist surface, stench, no access to pool/shower	Old faeces + urine present, ground kept dry, manual showering	Only recent faeces + urine, dry ground, short baths	Clean and dry surface, regular baths	Clean and dry surface available, free choice of water, mud and dust baths
**Environmental noise quality**	Direct vicinity to traffic, loud speaker system, large crowds	Intermediate of 0 and 2	Occasional traffic or small crowds, no electronic noise	Intermediate of 2 and 4	No noise except natural sounds
**Shelter**	Concrete ground, no sunlight/rain protection	Intermediate of 0 and 2	Dirt ground with medium shelter possibility (e.g single tree)	Intermediate of 2 and 4	Natural ground with sufficient and adequate shelter options
**Naturalness**	Urban environment	Intermediate of 0 and 2	Natural environment surroundings but direct contact only with artificial structures	Intermediate of 2 and 4	Fully based in natural environment
**Social interaction**	Solitary - no visual contact with conspecifics	Visual but no tactile contact	Tactile contact but no social grouping	Small social grouping possible	Complete free interaction with creation of social network
**Diet quality**	Inadequate amounts (<75kg/1000kg BDW) and limited variety	Adequate amounts but limited variety and quality	Adequate amounts, good variety and quality, always food available, limited water access	Adequate human selected variety, ad-libitum water and food	Sufficient natural food sources, free choice of consumption
**Entertainment intensity**	Show or intense use for other entertainment purposes	Undemanding shows once per day, trekking	Trekking without saddle or Be-a-Mahout program	No entertainment but strong interaction	Clear No-Entertainment policy
**Animal management**	No welfare understanding, inappropriate usage of ankhus, visible wounds on elephants, elephants constantly saddled, no vet treatments	Minimum welfare understanding, strong use of ankhus, treatment only through annual or bi-annual vet visits, elephants constantly saddled	Moderate welfare understanding, restricted use of ankhus only for required situations, call or transport to vet, no saddle unless needed	Good welfare understanding, sincere attempts to improve welfare standard, strong vet support	Very strong welfare understanding and focus on best situation for elephants, resident vet or strong vet support

B) Macaque venue score sheet

Category/Score	0	1	2	3	4
**Mobility**	Caged or short chain	Long chain/rope	Small enclosure / large cage with enrichment	Medium sized outdoor enclosure with vegetation	Large outdoor enclosure with natural vegetation
**Environmental noise quality**	Direct vicinity to traffic, loud speaker system, large crowds	Intermediate of 0 and 2	Occasional traffic or small crowds of visitors, no electronic noise	Intermediate of 2 and 4	No noise except natural sounds
**Shelter + Hygiene**	Concrete / mesh wire ground, no sunlight/rain protection, unhygienic (garbage, faeces)	Intermediate of 0 and 2	Dirt ground, shelter possibility (e.g single tree), moderately clean	Intermediate of 2 and 4	Natural ground, sufficient and adequate shelter options, regular cleaning
**Naturalness**	Urban environment	Intermediate of 0 and 2	Natural environment surroundings but direct contact only with artificial structures	Intermediate of 2 and 4	Fully based in natural environment
**Social interaction**	Solitary - no visual contact with conspecifics	Visual but no tactile contact with conspecifics	Tactile contact but no social grouping	Small social grouping possible	Complete free interaction with creation of social network
**Diet quality**	Inadequate amounts and limited variety	Adequate amounts but limited variety and no free water access	Adequate amounts, good variety, regularly fed, limited water access	Adequate human selected food variety, ad-libitum water and food	Sufficient natural food sources, free choice of consumption
**Entertainment intensity**	Regular circus show or intense use for other purposes	Undemanding shows once daily	Petting or feeding but no shows	No entertainment but strong visitor interaction	No entertainment and no interaction with visitors
**Animal management**	No welfare understanding, malnourished animals, long working hours, strong commercial interest	Intermediate of 0 and 2	Moderate welfare understanding, attempts to create a better situation for macaques, limited working hours	Good welfare understanding, sincere attempts to improve welfare standards, e.g. with environmental enrichments	Very strong welfare understanding and focus on best situation for macaques, resident vet or strong vet support

C) Tiger venue score sheet

Category/Score	0	1	2	3	4
**Mobility**	Caged or short chain	Long chain/rope	Small enclosure / large cage with enrichment	Medium sized outdoor enclosure with vegetation	Large outdoor enclosure with natural vegetation
**Environmental noise quality**	Direct vicinity to traffic, loudspeaker system, large crowds	Intermediate of 0 and 2	Occasional traffic or small crowds of visitors, no electronic noise	Intermediate of 2 and 4	No noise except natural sounds
**Shelter + Hygiene**	Concrete ground, no sunlight/rain protection, unhygienic (garbage, faeces)	Intermediate of 0 and 2	Dirt ground, shelter possibility (e.g single tree), moderately clean	Intermediate of 2 and 4	Natural ground, sufficient and adequate shelter options, regular cleaning
**Naturalness**	Urban environment	Intermediate of 0 and 2	Natural environment surroundings but direct contact only with artificial structures	Intermediate of 2 and 4	Fully based in natural environment
**Social interaction**	Overcrowding	Intermediate of 0 and 2	Group housing with individual retreats	Intermediate of 2 and 4	Completely free choice of interaction or seclusion
**Diet quality**	Inadequate amounts and limited variety	Adequate amounts but limited variety and no free water access	Adequate amounts, good variety, regularly fed, limited water access	Adequate human selected food variety and amount, ad-libitum water	Sufficient natural food sources, free choice of consumption
**Entertainment intensity**	Regular circus show or intense use for other purposes	No show but photo opps/ cub feeding/ petting	No entertainment but strong interaction	Intermediate of 2 and 4	No entertainment and no interaction with visitors
**Animal management**	No welfare understanding, malnourished animals, long working hours, strong commercial interest	Intermediate of 0 and 2	Moderate welfare understanding, attempts to create a better situation for tigers, limited working hours	Good welfare understanding, sincere attempts to improve welfare standards, e.g. environmental enrichment	Very strong welfare understanding and focus on best situation for tigers, resident vet or strong vet support
