# Supplementary material for: Asian Elephant (Elephas maximus), Pig-Tailed Macaque (Macaca nemestrina) and Tiger (Panthera tigris) Populations at Tourism Venues in Thailand and Aspects of Their Welfare
Source: PLoS One. 2015 Sep 25;10(9):e0139092. doi: 10.1371/journal.pone.0139092 (PMC4583339; doi:10.1371/journal.pone.0139092)
Supplement: S2 Table — These data points in conjunction with direct observations and photographic records were with the score sheet to estimate a welfare score for each venue. (PDF) [file pone.0139092.s002.pdf]

S2 Table: List of data points and possible answers collected from each venue during the assessment visits. These data points in conjunction with direct observations and photographic records were with the score sheet to estimate a welfare score for each venue.

**Category**	**Values**
**Visitor numbers**	<50; 50-200; 201-500; >500
**Visitor number source**	Venue staff; 1 hour monitoring; Personal estimate
**Education**	No education; Basic; Comprehensive
**Education content**	Species information; Conservation; Animal welfare; Cultural use; Other
**Venue established**	> 5 years; ≤ 5 years; Unknown
**Communicated animal welfare awareness**	Strong; Moderate; Slight; None
**Veterinary healthcare**	Vet on site; Contracted external vet; By owner; No treatments; Unknown
**Observed behavioural problems**	Ratio of animals at one venue displaying behavioural anomalies: > 75% of animals; 50-75% of animals; 25-49% of animals; < 25% of animals; No animals
**Types of behavioural problems**	Pacing; Weaving; Self-mutilation; Inactive; Hair-plucking; Aggression; Teeth-grinding Licking; Other
**Observed health problems**	Ratio of animals at one venue displaying health problems: >75% of animals; 50-75% of animals; 25-49% of animals; <25% of animals; No animals
**Types of health problems**	Underweight; Overweight; Skin problem; Wound; Nail cracks; Toe abscesses; Limping; CNS; Other
**Enclosure/cage size**	1-10sqm; 10-50sqm; 50-200sqm; 200-2;000sqm; 2;000-10;000sqm; >10;000sqm
**Shelter type**	No shelter; Single tree; Natural dense canopy; Mesh net roof; Fixed roof
**Shelter floor quality**	Concrete; Mesh wire; Natural ground
**Environment quality**	Urban/Town; Rural; Field; Forest
**Social interaction of animals**	None; Visual/Audio; Tactile + Visual/Audio; Kept in social group
**Feed type (Elephants)**	Pineapple fruit; Pineapple leaves; Banana fruit; Banana leaves/stem; Bamboo; Cucumber; Corn; Grass/Hay; Sugarcane; Processed food; Other 1:; Other 2:; Unknown
**Feed amount (Elephants)**	<75kg per 1000kg BDW; 75-100kg per 1000kg BDW; Ad libitum; Unknown
**Drinking Water**	Only sporadic; Regularly supplied; Free access
**Washing (Elephants)**	No bath/showers; Water hose shower; Supervised bath; Free bath at multiple times
**Ownership (Elephants)**	Venue owned; Mahout owned; Rented; Unknown
**If venue owned - how acquired**	Monetary purchase; Alternative livelihood exchange; Rescue/Confiscation/Donation; Unknown
**Entertainment**	Trekking/Rides; Show; Washing/Bathing; Feeding; Be-a-Mahout; Petting; Photo-opportunities; Cub feeding; Clear policy against use of animals in entertainment
**# of animals used for entertainment**	Number (#) of animals used for: Show; Photo opportunities; Cub feeding; Petting/Posing; Riding; Be-a-Mahout
**Shows per day**	1; 2-3; >3
**Trekking**	Uncoated saddle rope; Insufficient cushioning; > 3 people riding; Through natural habitat; Through village/town/park; ONLY without saddle
**Working hours**	>8 hours; 6-8 hours; <6 hours; no working hours
