# Supplementary material for: Asian Elephant (Elephas maximus), Pig-Tailed Macaque (Macaca nemestrina) and Tiger (Panthera tigris) Populations at Tourism Venues in Thailand and Aspects of Their Welfare
Source: PLoS One. 2015 Sep 25;10(9):e0139092. doi: 10.1371/journal.pone.0139092 (PMC4583339; doi:10.1371/journal.pone.0139092)
Supplement: S3 Table — (PDF) [file pone.0139092.s003.pdf]

S3 Table: Definitions of terms used in this study.

Term	Definition
**Chained husbandry**	Animals are restrained by chains (one-legged or two-legged ankle chains with lengths of less than 5m for elephants, collar-chains for macaques and tigers)
**Solitary husbandry**	Animals are kept for most of the day under conditions that do not allow opportunities for tactile interaction, such as grooming or social bounding
**Shelter**	Any natural or artificial structure that the animal has access to and that allows it to avoid exposure to direct sunlight and weather
**Pacing stereotypy**	Continuous back and forth walking along the same path for at least three repetitions
**Weaving stereotypy**	Continuously shifting weight from one body side to the other while standing in one place, often accompanied by swinging the head
**Self-mutilation stereotypy**	Self-inflicting injury and physical harm through biting of limbs or tails, or hitting the head against a wall
**Hair plucking stereotypy**	Continuous plucking of hair on limbs or other body parts, leading to bald patches or irritated skin
**Licking stereotypy**	Continuous licking of the same spot on a body part in absence of injury, leading to hair loss and irritated skin
**Other behaviour stereotypies**	Includes all other recognized stereotypic movements such as head bobbing (up and down movement of the head), spinning, bouncing or self-clasping
**Teeth grinding behaviour**	Animals are grinding their teeth, producing a notable sound
**Aggressive behaviour**	Animals display unusual amount of aggression towards conspecies, visitors or keepers, such as charging, showing teeth or hissing
**Irresponsive behaviour**	Animals that are awake do not react at all to events in the environment and do not interact with any object or animal for at least 5min
**Animal show attraction**	Animals are regularly made to perform various unnatural activities, such as balancing on two legs, riding tricycles, painting, or walking on tight-ropes within an arena for an audience
**Washing/bathing attraction**	Visitors are permitted to wash an animal under guidance by animal supervisors
**Photo opportunities attraction**	Visitors are allowed to pose for a photograph with restrained animals reserved for this purpose, e.g. tigers, orang-utans, macaques
**Cub feeding attraction**	Tiger cubs, previously removed from their mothers, are being presented to tourists to be fed with prepared milk formulation
**Be-a-mahout attraction**	Elephants are being assigned a tourist to handle it under supervision by a mahout for a half or full day
