# Supplementary material for: Asian Elephant (Elephas maximus), Pig-Tailed Macaque (Macaca nemestrina) and Tiger (Panthera tigris) Populations at Tourism Venues in Thailand and Aspects of Their Welfare
Source: PLoS One. 2015 Sep 25;10(9):e0139092. doi: 10.1371/journal.pone.0139092 (PMC4583339; doi:10.1371/journal.pone.0139092)
Supplement: S4 Table — The table shows lowest scores for elephants and macaques for ‘Mobility’ and ‘Entertainment intensity’, while for tiger venues lowest scores resulted for ‘Naturalness’ and also ‘Entertainment intensity’. Means are given purely as orientation as the data is ordinal. However, due to the approximate interval-type nature of the used scale, means may give valuable additional insight. (PDF) [file pone.0139092.s004.pdf]

S4 Table: Medians of the score sheet scores by husbandry factor and species kept at the venues. The table shows lowest scores for elephants and macaques for 'Mobility' and 'Entertainment intensity', while for tiger venues lowest scores resulted for 'Naturalness' and also 'Entertainment intensity'. Means are given purely as orientation as the data is ordinal. However, due to the approximate interval-type nature of the used scale, means may give valuable additional insight.

Score sheet category	Score sheet scores - Elephant venues[1]			Score sheet scores - Tiger venues[1]			Score sheet scores - Macaque venues[1]		
	n	Median	Mean	n	Median	Mean	N	Median	Mean
Animal management	105	1	1.124	10	1	1.3	21	1	1.238
Diet quality	105	2	1.924	10	1.5	1.6	21	1	1.381
Entertainment intensity	105	1	0.933	10	1	1	21	2	0.476
Environmental noise quality	105	2	2.152	10	1	1.1	21	1	1.429
Mobility	105	1	0.819	10	1.5	1.6	21	2	0.619
Naturalness	105	2	1.667	10	1	0.8	21	1	0.952
Social interaction	105	2	1.848	10	1.5	1.5	21	1	1.143
Shelter[2]	105	2	1.886	-	-	-	-	-	-
Hygiene[2]	105	2	2.076	-	-	-	-	-	-
Shelter and hygiene[3]	-	-	-	10	1	1.6	21	1	1.429

[1]Score sheet scores were between 0 and 4, where 0 reflected lowest and 4 highest animal welfare standards

[2]Shelter and hygiene were assessed independently in elephants

[3]Shelter and hygiene assessment was combined in tigers and macaques
